# A prodrug strategy for the *in vivo* imaging of aldehyde dehydrogenase activity†

**DOI:** 10.1039/d2cb00040g

**Published:** 2022-03-11

**Authors:** Raul Pereira, Renée L. Flaherty, Richard S. Edwards, Hannah E. Greenwood, Adam J. Shuhendler, Timothy H. Witney

**Affiliations:** School of Biomedical Engineering and Imaging Sciences, King's College London, St. Thomas’ Hospital London SE1 7EH UK tim.witney@kcl.ac.uk +44 (0)20 7188 7188, ext. 883496; Department of Chemistry & Biomolecular Sciences, University of Ottawa Ottawa ON Canada; University of Ottawa Heart Institute Ottawa ON Canada

## Abstract

Therapy resistance is one of the biggest challenges facing clinical oncology. Despite a revolution in new anti-cancer drugs targeting multiple components of the tumour microenvironment, acquired or innate resistance frequently blunts the efficacy of these treatments. Non-invasive identification of drug-resistant tumours will enable modification of the patient treatment pathway through the selection of appropriate second-line treatments. Here, we have designed a prodrug radiotracer for the non-invasive imaging of aldehyde dehydrogenase 1A1 (ALDH1A1) activity. Elevated ALDH1A1 activity is a marker of drug-resistant cancer cells, modelled here with matched cisplatin-sensitive and -resistant human SKOV3 ovarian cancer cells. The aromatic aldehyde of our prodrug radiotracer was intracellularly liberated by esterase cleavage of the geminal diacetate and specifically trapped by ALDH through its conversion to the charged carboxylic acid. Through this mechanism of action, ALDH-specific retention of our prodrug radiotracer in the drug-resistant tumour cells was twice as high as the drug-sensitive cells. Acylal masking of the aldehyde afforded a modest protection from oxidation in the blood, which was substantially improved in carrier-added experiments. *In vivo* positron emission tomography imaging of tumour-bearing mice produced high tumour-to-background images and radiotracer uptake in high ALDH-expressing organs but was unable to differentiate between drug-sensitive and drug-resistant tumours. Alternative strategies to protect the labile aldehyde are currently under investigation.

## Abstract 2

An *in vivo* aldehyde protection strategy for the non-invasive imaging of ALDH1A1 using a fluorine-18 labelled substrate.

## Graphical abstract

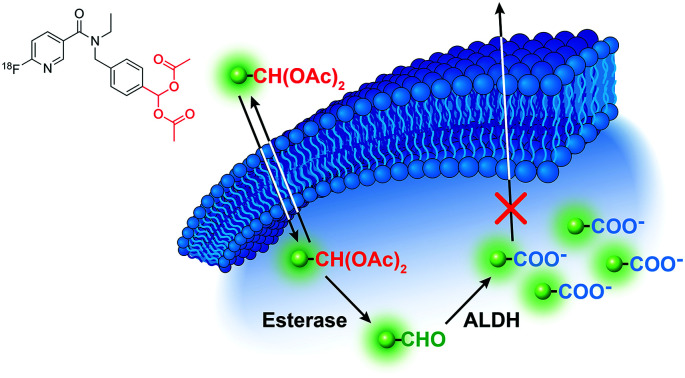

## Introduction

Aldehyde dehydrogenases (ALDHs) are a family of NAD(P)-dependant enzymes that catalyse the oxidation of aldehydes to carboxylic acids.^1^ Endogenous aldehydes can be produced as a result of metabolism of amino acids, alcohols, lipids and vitamins, while exogenous aldehydes can derive from the metabolism of cytotoxic drugs and environmental factors.^2–6^ Currently 19 ALDH isoforms have been characterised in humans,^7^ and ALDH1 isoforms (ALDH1A1, ALDH1A2, ALDH1A3) are considered to be of particular interest due to their role in the detoxification of anti-cancer drugs.^8^

ALDH1A1 expression has been correlated with poor overall survival in a range of cancers, including leukemia, lung, liver, pancreatic, breast, colorectal and ovarian cancer.^8^ Resistance to chemotherapy represents a significant challenge in the treatment of ovarian cancer, with a high proportion of women ultimately succumbing to recurrent disease.^9^ Tumour recurrence arises from drug-resistant tumour cells able to repopulate the tumour niche and seed new lesions. These cells have been termed cancer stem cells (CSCs), and are characterised by high ALDH1A1 expression, alongside other biomarkers such as CD133 and CD44.^10,11^ A programme of transcriptional, metabolic, and immunosurveillance pathways further contribute to therapy resistance.^12^ Extensive effort has focused on the creation of ALDH1A1-targeted therapies, with some showing success in reducing proliferation and expression of stemness markers in CSC-enriched ovarian cancer *in vitro* models,^13^ as well as synergising with cisplatin treatment to improve efficacy.^14^ Moreover, inhibition of ALDH1A1 in animal models of ovarian cancer has proven an attractive therapeutic strategy for CSC depletion and the prevention of recurrence.^15^

Given the causal link between ALDH1A1 expression and chemoresistance in some cancers, the identification of high ALDH1A1-expressing tumour represents a clinical challenge that if solved, could significantly improve patient outcomes. Imaging these tumours using ALDH1A1 expression as a marker of chemoresistance could inform therapeutic interventions and offer the chance to tailor bespoke patient treatments.^16^ Current imaging strategies for evaluating ALDH activity have to the most part been restricted to fluorescence-based assays in isolated cells.^17–20^ Recently, however, Anorma *et al.* have used the fluorescent AlDeSense probe to image tumours *in vivo* following direct intra-tumoral administration.^17^ Despite these commercially-available imaging agents being widely-adopted for the isolation of ALDH-positive cells in cell culture, the poor tissue penetration of the fluorescent signal and requirement for direct tumour injection currently limits their *in vivo* utility. In order to circumvent these inherent limitations, us and others have proposed the use of positron emission tomography (PET) as an alternative to fluorescence-based imaging.^21,22^ We have recently reported that [^18^F]*N*-ethyl-6-(fluoro)-*N*-(4-formylbenzyl)nicotinamide – [^18^F]1 – has excellent affinity and isozyme selectivity for ALDH1A1 in cancer cells but is rapidly oxidised to its corresponding carboxylic acid in the blood when administered at tracer doses.^21^ Here, we employed an enzyme-cleavable prodrug strategy to increase the blood stability of our ALDH1A1-targeted radiotracer. Protecting the metabolically labile aldehyde from oxidation maintained ALDH1A1 specificity, producing positive tumour-to-background images in mouse models of ovarian cancer.

## Results and discussion

### Synthesis of a prodrug radiotracer for ALDH imaging

We chose to use a geminal diacetate to protect the aldehyde on [^18^F]1 to create [^18^F](4-((*N*-ethyl-6-fluoronicotinamido)methyl)phenyl)methylene diacetate [^18^F]2. This protecting group was chosen as it is stable to acid and mildly basic conditions, but importantly is susceptible to enzymatic cleavage *via* esterase activity to intracellularly liberate the free aldehyde. ALDH1A1 is known to exhibit esterase activity^23^ and we envisaged that following passive diffusion into the cell, the diacetate would be cleaved to furnish [^18^F]1. ALDH-mediated oxidation of [^18^F]1 to the carboxylate [^18^F]3 would subsequently result in intracellular trapping of the radiolabelled product as a result of its negative charge.

**Scheme 1:**
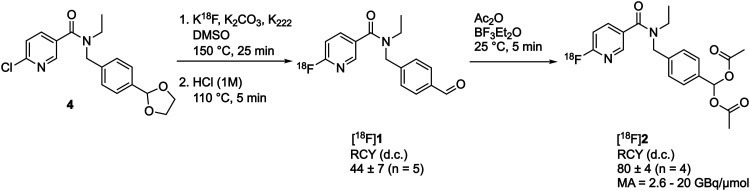
Radiosynthesis of [^18^F]2. RCY, radiochemical yield; d.c., decay-corrected; MA, molar activity.

The synthetic strategy to access [^18^F]2 first required synthesis of the aldehyde [^18^F]1, which could be subsequently activated for reaction with acetic anhydride. A nucleophilic aromatic substitution (S_N_Ar) was performed on *N*-(4-(1,3-dioxolan-2-yl)benzyl)-*N*-ethyl-6-chloronicotinamide 4 with [^18^F]KF/K_222_ in DMSO at 150 °C for 25 min followed by an acid mediated acetal-cleavage step to furnish [^18^F]1 in 44 ± 7% radiochemical yield (RCY; *n* = 5; for further details see Fig. S1 and S2, ESI†). The other major radioactive component of this two step, one pot reaction was accounted for as unreacted [^18^F]fluoride from the radiofluorination reaction, as has been described for this synthesis previously.^21^ [^18^F]1 was stirred with acetic anhydride and BF_3_·Et_2_O at room temperature for 5 min to produce [^18^F]2 in 80 ± 4% isolated RCY (*n* = 4) with a molar activity of up to 20 GBq μmol^−1^ (Scheme 1). Reformulation of [^18^F]2 in PBS ready for biological evaluation was achieved with high radiochemical purity (>98%) and stability (>95% radiochemical purity after for 4 h; see Fig. S3, ESI†).

### [^18^F]2 is cleaved by esterases and provides a readout of ALDH activity in cancer cells

**Scheme 2:**
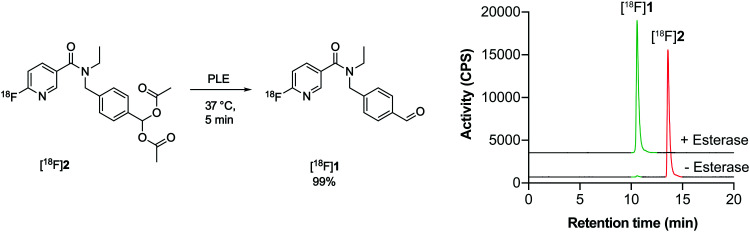
PLE cleavage of [^18^F]2 (retention time, 13.6 min; red) to furnish [^18^F]1 (retention time 10.6 min; green).

With a radiotracer candidate in-hand we assessed whether [^18^F]2 was an esterase substrate. Treatment of [^18^F]2 with porcine liver esterase (PLE) at 37 °C for 5 min resulted in the liberation of the desired aldehyde [^18^F]1 with complete conversion of [^18^F]2 to [^18^F]1, validating this protection strategy (Scheme 2).

**Fig. 1:**
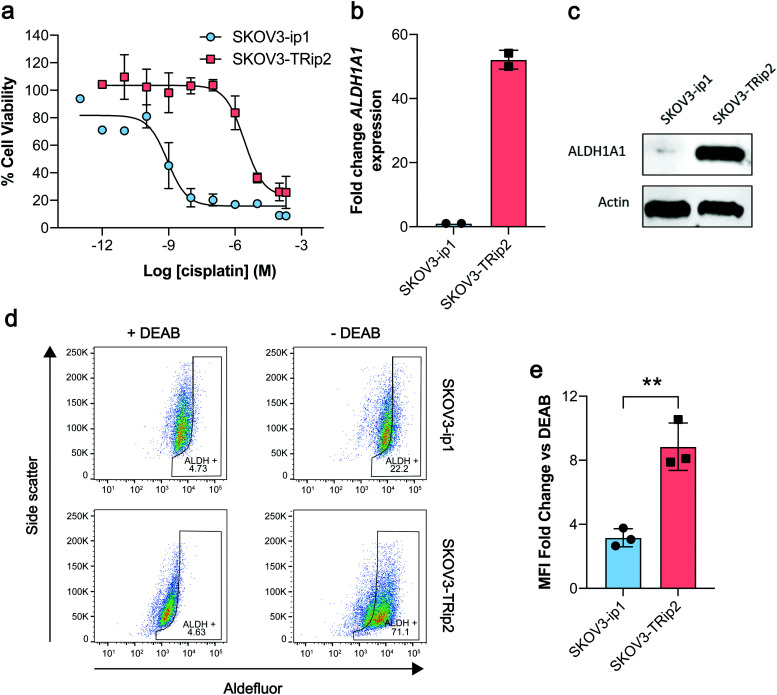
Characterisation of matched cisplatin sensitive (SKOV3-ip1) and resistant (SKOV3-TRip2) ovarian cancer cell lines. (a) Cisplatin-induced growth inhibition in cells using an MTT assay 72 h post treatment (*n* = 3–5). (b) ALDH1A1 mRNA expression in SKOV3-ip1 and SKOV3-TRip2 cells (*n* = 2). (c) ALDH1A1 protein expression in cells. (d) ALDH-specific retention of the Aldefluor reagent in the presence and absence of the ALDH inhibitor *N*,*N*-diethyl amino benzaldehyde (DEAB). (e) Ratio of Aldefluor median fluorescence intensity (MFI) in DEAB and vehicle-treated cells (*n* = 3). **, *P* < 0.01. Data are means ± SD.

Having shown that [^18^F]2 successfully undergoes esterase-mediated cleavage to the aldehyde [^18^F]1, we assessed whether [^18^F]2 could provide a readout of ALDH activity in tumour cells grown in culture. For this we chose two human ovarian cancer lines, SKOV3-ip1 and SKOV3-TRip2, that had been previously passaged intraperitoneally in mice to select for cells with enhanced tumourgenicity.^10^ The SKOV3-TRip2 cell line was generated through progressive exposure to paclitaxel,^24^ whereas the SKOV3-ip1 cell line remained untreated. Similar to previous findings with paclitaxel,^10^ resistance to cisplatin was increased by over three orders of magnitude in SKOV3-TRip2 cells compared to SKOV3-ip1 (EC_50_, 0.88 nM *vs.* 2.6 μM, respectively; *n* = 3–5; Fig. 1a). In SKOV3-TRip2 cells, ALDH1A1 mRNA (Fig. 1b) and protein expression (Fig. 1c) were substantially increased in comparison to SKOV3-ip1 cells. Importantly, ALDH activity, as measured by the Aldefluor assay,^25^ was also significantly increased in SKOV3-TRip2 cells compared to SKOV3-ip1 (*n* = 3; *P* < 0.01; Fig. 1d and e).

**Fig. 2:**
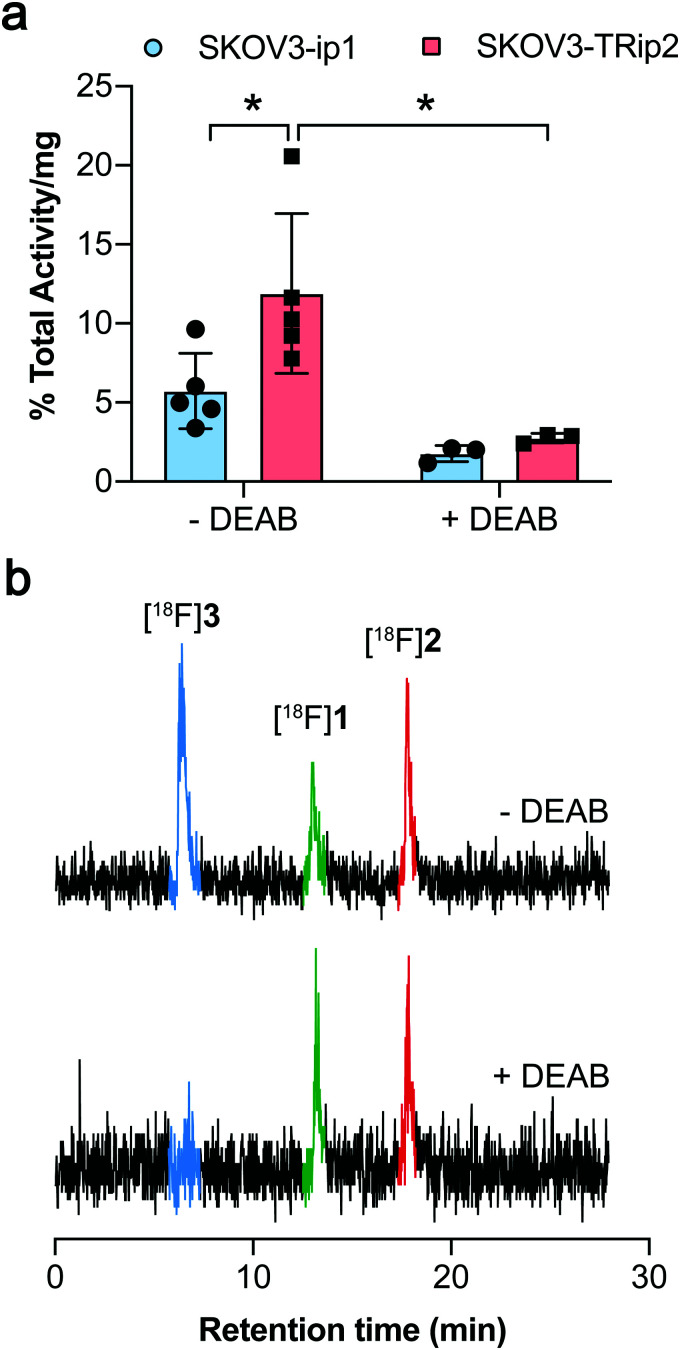
Cleavage and cellular retention of protected [^18^F]2 in ovarian cancer cells. (a) Cell-associated radioactivity in SKOV3-ip1 and SKOV3-TRip2 cells following incubation with [^18^F]2 for 20 min in the presence and absence of DEAB (30 μM). Data are means ± SD. (b) Radio-HPLC chromatograms from SKOV3-TRip2 cell lysates following 20 min incubation of [^18^F]2 (red peak) with (bottom), or without DEAB treatment (top). The green peak corresponds to the aldehyde [^18^F]1, with the blue peak corresponding to the carboxylate [^18^F]3. *, *P* < 0.05. Data are means ± SD.

ALDH-specific cellular retention of [^18^F]2 was examined 20 min after radiotracer addition to SKOV3-ip1 and SKOV3-TRip2 cell lines, as previously optimised with [^18^F]1.^21^ [^18^F]2 was rapidly taken up and trapped in the high ALDH1A1-expressing SKOV3-TRip2 cells, which was ∼2-fold higher than in SKOV3-ip1 cells, at 11.9 ± 5.1% activity per mg protein and 5.7 ± 2.4% activity per mg protein, respectively (*n* = 5; *P* < 0.05; Fig. 2a). Intracellular liberation of a reactive aldehyde may allow for its conjugation with cellular nucleophiles, resulting in trapping of the substrate independent of ALDH activity. To assess ALDH-specific trapping of [^18^F]2, cells were co-incubated with the pan-ALDH inhibitor *N*,*N*-diethyl amino benzaldehyde (DEAB), which significantly reduced intracellular radioactivity in SKOV3-TRip2 by 77%, falling to 2.7 ± 0.3% activity per mg protein (*n* = 3; *P* < 0.05), ruling out non ALDH-mediated mechanisms of trapping. To confirm the identity of the intracellular radioactive species present, we performed radio-HPLC analysis of SKOV3-TRip2 cell lysates. 20 min after radiotracer addition, enzymatic deprotection of [^18^F]2 to [^18^F]1 by intracellular (human) esterases and subsequent conversion to the corresponding carboxylic acid [^18^F]3 was observed, confirmed using non-radioactive fluorine-19 isotopologues (see ESI† for details). With DEAB treatment of cells, cleavage to [^18^F]1 occurred as expected, however, subsequent oxidation to [^18^F]3 was prevented (Fig. 2b). Taken together, this suggests that intracellular production of the carboxylate [^18^F]3 is mediated by ALDH, the enzymatic activity of which determines total intracellular radiotracer retention. [^18^F]2 therefore provides a sensitive measure of ALDH activity in tumour cells.

### Aldehyde protection and carrier-added formulation improve radiotracer blood stability

**Fig. 3:**
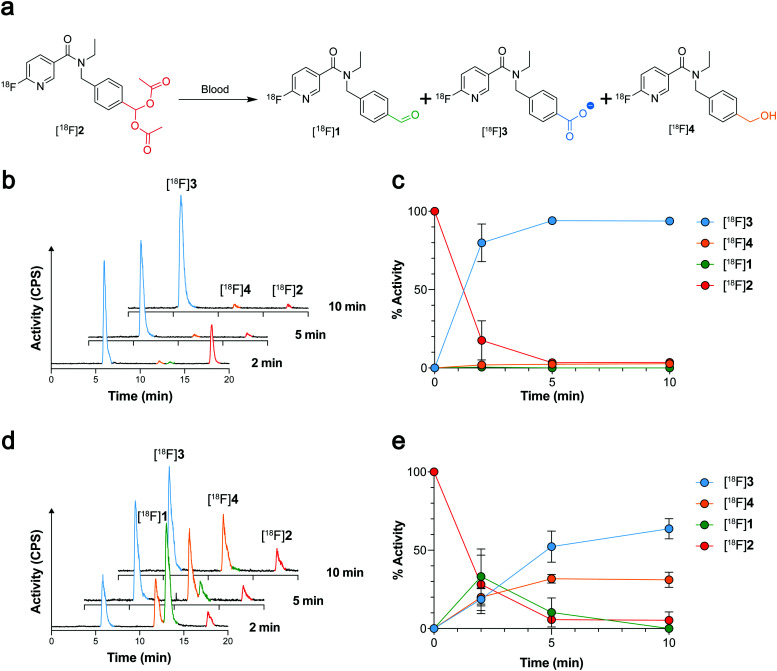
Blood metabolism of [^18^F]2. (a) Chemical structures of the products formed following incubation of [^18^F]2 in mouse blood. (b) Radio-chromatograms showing changes to the composition of radioactive metabolites following incubation of high MA (8.4 ± 6.9 GBq μmol^−1^) [^18^F]2 (red) in mouse blood at 2, 5 and 10 min. (c) Time course changes in radiolabelled metabolite composition following incubation of high MA [^18^F]2 (8.4 ± 6.9 GBq μmol^−1^; red) in mouse blood. Data are means ± SD (*n* = 3–6). (d) Radio-chromatograms showing changes to the composition of radioactive metabolites following incubation of low MA (0.11 ± 0.002 GBq μmol^−1^) [^18^F]2 (red) in mouse blood at 2, 5 and 10 min. (e) Time course changes in radiolabelled metabolite composition following incubation of low MA [^18^F]2 (0.11 ± 0.002 GBq μmol^−1^; red) in mouse blood. Data are means ± SD (*n* = 3 biological repeats).

Given that [^18^F]1 had previously undergone complete oxidation when incubated for 2 min in blood,^21^ likely due to high ALDH1 expression in erythrocytes,^26^ we next examined whether geminal diacetate protection afforded improved blood stability. Whilst [^18^F]2 was still present in murine blood at 2 min, rapid cleavage and oxidation to the carboxylate [^18^F]3 was observed (80 ± 12% activity; *n* = 6), increasing to 94 ± 1% activity (*n* = 3) at 5 min and negligible amounts of the aldehyde [^18^F]1 and alcohol [^18^F]4 seen (Fig. 3a–c and Fig. S4, ESI†). Interestingly when the molar activity (MA) of [^18^F]2 was lowered from 8.4 ± 6.9 GBq μmol^−1^ (0.36 nmol 2 injected) to 0.11 ± 0.002 GBq μmol^−1^ (25 nmol 2 injected) its blood metabolism was noticeably altered (Fig. 3d and e). With low MAs, the aldehyde [^18^F]1 was now the predominant species at early time points (33 ± 18% activity at 2 min; *n* = 3). Whilst esterase-mediated cleavage of [^18^F]2 occurred at similar rates to high MA preparations, oxidation of [^18^F]1 to the carboxylate was substantially reduced over the time course. Indeed, enough [^18^F]1 remained in the blood at low MAs for its reduction to the alcohol [^18^F]4 to occur, albeit at a slower rate of conversion than to the carboxylate [^18^F]3.

The amount of carrier-added 2 requires careful calibration. On one-hand, we have shown that decreasing MA prevents blood oxidation of the aldehyde, maintaining a sufficient pool of the ALDH substrate. However, a low MA has the potential to block ALDH-mediated oxidation of [^18^F]1 due to increased competition at the enzyme's active site. To test the effect of MA on SKOV3-TRip2 cell uptake, we incubated [^18^F]2 with varying concentrations of non-radioactive 2. A decrease in MA proportionally decreased SKOV3-TRip2 [^18^F]2 cell uptake. High radiotracer uptake, however, was still present with a MA of 0.11 GBq μmol^−1^ (Fig. S5, ESI†).

### *In vivo* PET imaging

**Fig. 4:**
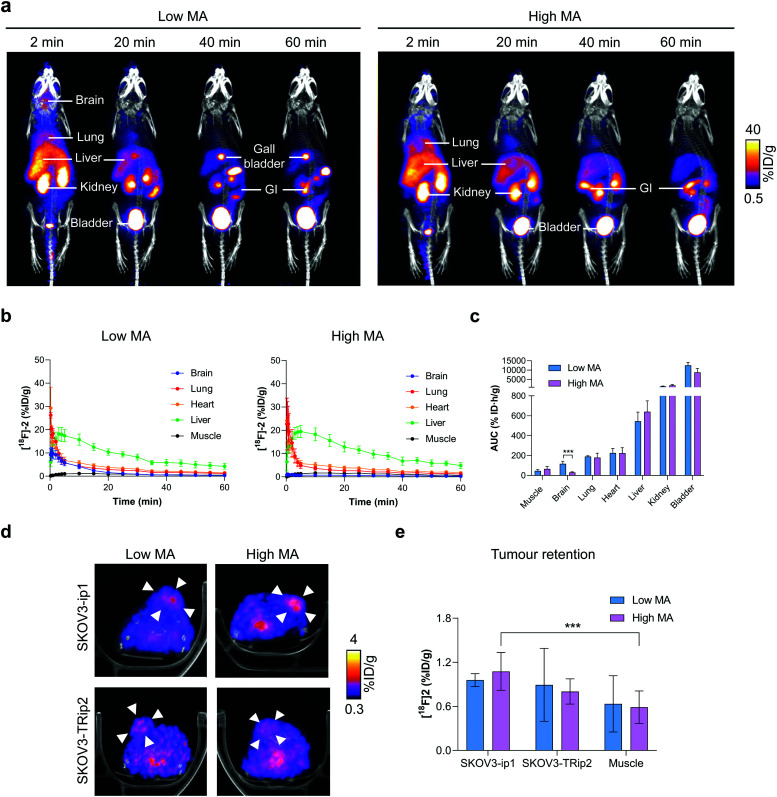
Dynamic PET/CT imaging of [^18^F]2 in ovarian cancer tumour-bearing mice. (a) Representative time course PET/CT maximum intensity projections of [^18^F]2 in balb/c nu/nu mice at low and high molar activities. Uptake in key organs are illustrated. GI, gastrointestinal tract. (b) Time *versus* radioactivity curves of major organs of interest normalised to the percentage injected activity. Data expressed as the mean ± standard deviation. *n* = 3–7 animals per group. (c) Area under the time activity curve for organs with substantial levels of [^18^F]2 uptake. *, *P* < 0.05; ***, *P* < 0.001. (d) Representative axial PET/CT images (50–60 minutes summed activity) from a dynamic 60 minutes scan following the injection of [^18^F]2 at low and high MA in balb/c nu/nu mice bearing either SKOV3-ip1 or SKOV3-TRip2 subcutaneous tumours. Arrowheads indicate the tumour, identified from the CT image. (e) Quantification of [^18^F]2 tumour uptake 50–60 min post injection. *n* = 3–7.

Given that [^18^F]2 can assess ALDH activity in SKOV3-ip1 and SKOV3-TRip2 cells with high sensitivity and specificity, we next explored the ability of [^18^F]2 to detect ALDH activity in tumour-bearing mice. Dynamic [^18^F]2 PET/CT imaging was performed at low and high MAs to understand the effect of radiotracer metabolism on its pharmacokinetics and tumour retention. [^18^F]2 was characterised by rapid extraction from the blood and excretion through the urinary tract (Fig. 4a). At early time points, high [^18^F]2 uptake was evident in the lung and liver, with liver uptake peaking at 3 and 5 min for low and high MAs, respectively (18.2 ± 2.9% ID mL^−1^ and 19.8 ± 2.4% ID mL^−1^; Fig. 4b). Radiotracer retention in the brain was significantly higher with low MA [^18^F]2 compared to high MA, shown through the area under the time activity curve (AUC; 117.1 ± 31.3% ID h g^−1^ and 35.4 ± 6.0% ID h g^−1^, respectively; *p* = 0.006; *n* = 3–7; Fig. 4c). High brain uptake at low MA is likely a product of increased blood concentrations of a blood brain barrier (BBB)-permeable [^18^F]1, or a consequence of efflux transporter (*e.g.* P-glycoprotein) saturation on the BBB, facilitating transient accumulation in the brain. Hepatobiliary excretion was evident by ∼20 min p.i., suggesting possible metabolism of the parent compound at this time point (Fig. 4a).

Representative axial PET/CT images of SKOV3-ip1 and SKOV3-TRip2 tumours at both high and low MAs are shown in Fig. 4d. Importantly, 20 min post-injection of [^18^F]2, positive SKOV3-ip1 tumour-to-muscle ratios >1.2 were observed, that continued to increase to 2.5 and 2.1 in SKOV3-ip1 tumours 50–60 min post-injection for low and high MA scans, respectively (Fig. S6a and b, ESI†). In high MA scans, 50–60 min after administration, retention of [^18^F]2 reached 1.1 ± 0.3% ID mL^−1^ in SKOV3-ip1 tumours compared to 0.5 ± 0.2% ID mL^−1^ in the muscle (*p* < 0.001; *n* = 7; Fig. 4e). There was no difference in retention of [^18^F]2 in SKOV3-ip1 and SKOV3-TRip2 tumours either at low (1.0 ± 0.1% ID mL^−1^ and 0.9 ± 0.5% ID mL^−1^, respectively; *p* = 0.8; *n* = 3) or high MAs (1.1 ± 0.3% ID mL^−1^ and 0.8 ± 0.2% ID mL^−1^, respectively; *p* = 0.06; *n* = 3–7). At high MAs, it is likely that the geminal diacetate only offers protection against blood oxidation during the first pass, limiting the ability of [^18^F]2 to differentiate between high and low ALDH-expressing tumours. Conversely, whilst addition of carrier-added 2 reduces ALDH-mediated blood oxidation, the unlabelled compound also competes with [^18^F]2 for tumour ALDH, thereby blocking production of [^18^F]3.

## Conclusions

In summary, we have developed a prodrug strategy for the non-invasive imaging of ALDH activity in animal models of human ovarian cancer. Here, acylals were employed as an *in vivo* cleavable protecting group for the aromatic aldehyde of a highly-specific ALDH1A1 radiotracer. In cell culture, the aldehyde [^18^F]1 was intracellularly liberated by esterases and trapped by ALDH1A1 through conversion to the carboxylic acid [^18^F]3. Consequently, high radiotracer retention was measured in culture in the drug-resistant SKOV3-TRip2 tumour cells which have high ALDH1A1 expression. Pharmacological inhibition of ALDH additionally reduced tumour cell retention of our radiotracer [^18^F]2.

*In vivo*, the acylal group at trace levels was rapidly cleaved in the blood, the extent of which was substantially reduced through co-injection of the isotopologue [^19^F]2. PET imaging of [^18^F]2 in ovarian tumour-bearing mice at high and low molar activities revealed tumour-specific uptake, but was unable to distinguish between high and low ALDH-expressing tumours. Future studies will explore alternative methods to increase the blood half-life of [^18^F]2 through carrier-added studies using compounds that are substrates for esterases but not ALDH1A1, by altering serum protein binding, and fine-tuning the protecting group's chemical properties for improved blood stability.

## Materials and methods

### Chemistry: general procedures and instrumentation

Commercially available starting materials were purchased from Sigma-Aldrich, Alfa Aesar, TCI Europe, and Apollo Scientific and were used without further purification. Solvents were obtained from Sigma-Aldrich; unless stated otherwise, reagent grade solvents were used for reactions and column chromatography. Reaction progress was monitored by thin layer chromatography (TLC) on aluminium sheets coated with silica gel 60 F254 (Merck Millipore) and detection was carried out using UV light (325 nm and 254 nm) and/or chemical solutions. Crude reaction mixtures were purified by automated flash column chromatography (Biotage Isolera One).

^1^H, ^13^C, and ^19^F Nuclear Magnetic Resonance (NMR) spectra were recorded on a Bruker Avance 400 room temperature. ^13^C NMR experiments were proton decoupled. ^1^H and ^13^C NMR spectra are reported relative to the internal reference of the relative deuterated solvent. Chemical shifts (*δ*) are reported in ppm and coupling constants (*J*) are given in Hertz (Hz). Multiplicity is described with (s): singlet, (d): doublet, (t): triplet and (q): quadruplet. High resolution mass spectrometry data were recorded on a Thermo Finnigan MAT900xp (CI, EI), Agilent 6510 QTOF (ESI), Waters LCT Premier spectrometers (ESI), or Thermo Exactive mass spectrometer with an Orbitrap (ESI, APCI).

#### (4-((*N*-Ethyl-6-fluoronicotinamido)methyl)phenyl)methylene diacetate, (2)

A round bottomed flask (10 mL) was charged with *N*-ethyl-6-fluoro-*N*-(4-formylbenzyl)nicotinamide^1^ (0.15 g, 0.52 mmol) and acetic anhydride (2 mL). The reaction was cooled to 0 °C and then BF_3_·Et_2_O (0.05 mL) was injected. The flask was stirred at room temperature for 3 hours after which the reaction was quenched with NaHCO_3_ (sat. aq.) (10 mL) and then extracted with ethyl acetate (25 mL). The ethyl acetate layer was rinsed with NaHCO_3_ (10 mL), water (10 mL), brine (10 mL) and then dried over MgSO_4_. The solvents were removed under reduced pressure to yield an oily residue which was purified *via* flash chromatography [silica gel Biotage Sfär (10 g), DCM wet-load, ethyl acetate/methylene chloride (0 : 100) to (20 : 80)] to yield (4-((*N*-ethyl-6-fluoronicotinamido)methyl)phenyl)methylene diacetate 2 as a an off-white solid (0.171 g, 85%). ^1^H NMR (400 MHz, DMSO-d_6_, 298 K): *δ* [ppm] = 1.04–1.12 (m, 3H, CH_2_C*H*_3_), 2.11 (s, 6H, OAc), 3.18–3.39 (m, 2H, C*H*_2_CH_3_), 4.52–4.71 (m, 2H, C*H*_2_), 7.20 (m, 2H, Ar–*H*), 7.45 (m, 1H, Ar–*H*), 7.48–7.50 (m, 2H, Ar–*H*), 7.55 (s, 1H, Ar–C*H*(OAc)_2_), 8.04–8.13 (m, 1H, Ar–*H*), 8.30–8.39 (m, 1H, Ar–*H*). Note: compound exists as mixture of rotamers. ^13^C NMR (100 MHz, DMSO-d_6_, 298 K): *δ* [ppm] = 13.6, 14.1, 20.5, 43.4, 46.6, 59.73, 88.9, 109.7 (d, *J*_F–C_ = 37 Hz), 126.7, 127.7, 131.0, 134.2, 139.3, 140.6 (d, *J*_F–C_ = 8 Hz), 145.5 (d, *J*_F–C_ = 15 Hz), 162.9 (d, *J*_F–C_ = 238 Hz), 167.5, 168.7. ^19^F NMR (375 MHz, DMSO-d_6_, 298 K): *δ* [ppm] = −67. HRMS (ESI) *m*/*z* [M + H]^+^: calc. for C_20_H_22_N_2_O_5_F: 389.1507: found 389.1495.

Compounds 1, 3 and 4 were prepared as previously described.^21^

### Radiochemistry: general procedures and instrumentation

[^18^F]Fluoride was produced by a GE PETrace cyclotron by 16 MeV irradiation of enriched [^18^O]H_2_O target, supplied by Alliance Medical Radiopharmacy Ltd (London, UK) or St. Thomas’ Hospital (London, UK) in approximately 3 mL of water. [^18^F]Fluoride was used without further purification. Radioactivity was measured in a CRC-25R dose calibrator (Capintec, Inc). The automated radiosynthesis platform used in the study was the GE FASTLab™. QMA light SepPak cartridge (WAT186004051) were purchased from Waters (Elstree, UK). C18 SepPak cartridges (WAT036805) were purchased from Waters (Elstree, UK) and were conditioned using EtOH (5 mL) and water (10 mL). The precursor 4 was synthesised according to previous reports.^21^ Analytical and semi-preparative RP-HPLC were performed with an Agilent 1200 HPLC system equipped with a 1200 Series Diode Array Detector and a Raytest GABI Star NaI(Tl) scintillation detector (energy window 400–700 keV). Isolated radiochemical yield (RCY) refers to the activity of the pure tracer isolated after HPLC divided by the initial activity of [^18^F]fluoride in [^18^O]H_2_O used for the labelling. RCYs are given decay corrected. Radiochemical purity refers to the proportion of the total radioactivity in the sample which is present as the desired radiotracer, as measured by radio-HPLC.^27^

### Automated radiosynthesis of [^18^F]1 on the GE FASTLab

The GE FASTLab™ was programmed to perform the following protocol using a custom cassette layout (Fig. S7, ESI†). The procedure was adapted from that previously reported by our group.^21^ [^18^F]Fluoride (1–2 GBq) was trapped on a QMA light SepPak cartridge and the ^18^O water eluted into a collection vial for recovery. Kryptofix carbonate solution [Kryptofix (8.0 mg, 21.2 μmol), potassium carbonate (1.1 mg, 8.0 μmol), acetonitrile (0.650 mL), and water (0.200 mL)] was taken up by syringe 1 and eluted through the QMA light SepPak cartridge into the reaction vessel. The [^18^F]fluoride/Kryptofix/carbonate mixture was azeotropically dried at 120 °C under a mixture of nitrogen pressure (200 mbar) and vacuum (−1000 mbar) using anhydrous acetonitrile (3 × 0.40 mL). The precursor 4 (10.0 mg, 28.8 μmol) was added to the reaction vessel as a solution in anhydrous DMSO (1 mL). Following heating at 150 °C for 25 min, the reaction temperature was lowered to 100 °C. Aqueous HCl (1 mL, 1 M) was added and the reaction mixture kept at 100 °C for 5 min. The reaction temperature was lowered to 70 °C, and a solution of ammonium acetate (3 mL, 20 mM) was added. After 5 min the reaction mixture was transferred into an external vial containing a solution of ammonium acetate (30 mL, 20 mM). Nitrogen was passed through the mixture for 20 s to encourage mixing. The crude product was extracted on to two C18 SepPak cartridges, using syringe 2 for loading in a stepwise manner. A slower loading was achieved by breaking down the syringe plunger movements into multiple programmed stop/start commands (12 × 5 second intervals) as reported previously.^28^ Washing with water (28 mL) was then performed in the same fashion. Both the extraction of [^18^F]1 and washing steps were performed by loading onto the female end of the cartridge. Syringe 3 was then used to pass the MeOH eluent (20 mL) through the two C18 SepPak cartridges in a ‘reverse elution’, passing in the opposite direction to which the C18 SepPak cartridges were loaded and washed. The product was eluted through an external switch valve for collection in a glass vial to give [^18^F]1 in 44 ± 7% RCY (*n* = 5) with >98% radiochemical purity as determined by radio-HPLC (Eclipse XDB-C18, 9.4 × 250 mm, 5 μm HPLC column at room temperature; solvent A: H_2_O (10 mM NH_4_OAc), solvent B: MeOH; flow rate: 3.5 mL min^−1^; UV detector: 254 nm; gradient: 37–75% B, 0–21 min; 75–95% B, 21–22 min; 95% B; 22–26 min; 95–37% B, 26–27 min).

### Manual radiolabelling of [^18^F]2

[^18^F]1 (250–400 MBq) in methanol was added to a 4 mL Wheaton V vial and the methanol removed at 70 °C under a flow of nitrogen. Azeotropic distillation of any residual water was conducted by addition of MeCN (2 × 300 μL) and evaporation of volatiles at 70 °C under a flow of nitrogen for 10 min. The Wheaton V vial was sealed and left to cool for 10 min before addition of acetic anhydride (300 μL) to the residue. BF_3_·Et_2_O (1 M in THF, 5 drops) was added to the solution and the reaction mixture was stirred at room temperature for 5 min. The reaction mixture was then taken up into a syringe and added dropwise to an ice cold solution of ammonium acetate (1.5 mL, 20 mM). To minimise hydrolysis of [^18^F]2 back to [^18^F]1 during this quenching step, the ammonium acetate solution was placed at room temperature 2 min before use and broken up to form a slurry. Methanol (500 μL) was added to the crude mixture and the resulting solution was purified by HPLC chromatography (Eclipse XDB-C18, 9.4 × 250 mm, 5 μm HPLC column at room temperature; solvent A: H_2_O (10 mM NH_4_OAc), solvent B: MeOH; flow rate: 3.5 mL min^−1^; UV detector: 254 nm; gradient: 37–75% B, 0–21 min; 75–95% B, 21–22 min; 95% B; 22–26 min; 95–37% B, 26–27 min, 5 mL injection loop, collection from 17.8 to 19.1 min) to give [^18^F]2 (150–250 MBq, 80 ± 4% RCY, *n* = 4). The isolated fraction (approx. 4 mL) was diluted with water (20 mL) before trapping of [^18^F]2 on a C18 SepPak cartridge by dropwise addition. The trapped product was washed with 10 mL water and eluted with 2 mL ethanol into a Wheaton V vial. The ethanol was removed at 70 °C under a flow of nitrogen before reformulation of [^18^F]2 in 800 μL PBS. HPLC analysis of the reformulated product was conducted to assess the radiochemical purity (>95%) and molar activity (8.4 ± 6.9 GBq μmol^−1^) of the reformulated product (Eclipse XDB-C18, 9.4 × 250 mm, 5 μm HPLC column at room temperature; solvent A: H_2_O (10 mM NH_4_OAc), solvent B: MeOH; flow rate: 3.5 mL min^−1^; UV detector: 254 nm; gradient: 37–75% B, 0–21 min; 75–95% B, 21–22 min; 95% B; 22–26 min; 95–37% B, 26–27 min, 100 μL injection loop, retention time = 16.78 min).

MA (expressed in GBq μmol^−1^) was measured using HPLC and was calculated using the equation:
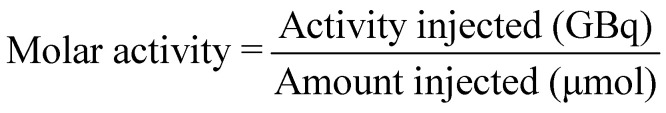
The amount injected was calculated using a calibration curve (Fig. S8, ESI†).

### Cell lines

Human ovarian cancer cell lines SKOV3-ip1 and SKOV3-TRip2 (a kind gift of Dr Anil Sood) were maintained in RPMI-1640 medium (Gibco, UK) supplemented with 10% fetal bovine serum (ThermoFisherScientific). SKOV3-TRip2 were maintained with the addition of 150 nM of paclitaxel, added every third passage.

### Drug sensitivity in cell culture

SKOV3-ip1 and SKOV3-TRip2 cells were plated at a density of 3 × 10^3^ and 6 × 10^3^ respectively in 96 well plates. The next day, cells were treated with increasing concentrations of paclitaxel (Adipogen, Life Sciences, UK) for 72 h. Complete media with no drug was used as a control. For cell viability measurements, cell culture media was removed and replaced with 100 μL of 0.5 mg mL^−1^ MTT dissolved in complete cell culture media. Plates were protected from the light and incubated for a further 4 h at 37 °C. The MTT solution was removed and replaced with 100 μL dimethyl sulfoxide (DMSO). Absorbance was measured at *λ* = 570 nm using a Multiskan FC Absorbance Plate Reader (THERMO-LABSYSTEMS). Viable cells were calculated as a percentage of the control group treated with no drug. Dose–response curves were generated and from them half-maximal growth inhibition (EC_50_) values were determined using GraphPad Prism (sigmoidal dose–response, fixed slope; v.8.0).

### RT-qPCR

RNA was extracted from SKOV3-ip1 and SKOV3-TRip2 cells using the RNeasy Kit (Qiagen, UK) and cDNA was synthesised using a Quantitect Reverse Transcription kit (Qiagen, UK) as per the manufacturer's instructions. A Rotor-Gene SYBR Green (Qiagen, UK) master mix was prepared according to the manufacturer's instructions using Quantitect Primer Assay for human *ACTB* and *ALDH1A1* (Qiagen, UK). Ct values were obtained using Rotor-Gene Q software. Change in expression was measured using the ΔΔCt method and expressed as relative expression *versus* the experimental control or an internal universal reference.

### ALDH activity

ALDH activity was measured by flow cytometry using the ALDEFLUOR kit (STEMCELL Technologies 01700) according to manufacturer's instructions. Cells were ALDEFLUOR stained at a concentration of 5 × 10^5^ cells per mL for 45 minutes at 37 °C in the presence or absence of 60 μM DEAB. The cell suspension was passed through a 35 μM filter and kept on ice prior to analysis. Flow cytometry was performed on a BD FACSMelody using FACS Chorus software (488 nm laser and 527/32 bandpass filter), followed by analysis with FlowJo. A minimum of 10 000 events were collected for each sample. Data were gated post-acquisition based on forward (FS) and side scattering (SS) profiles to include only single cell events and to exclude cellular debris.

### Esterase assay

To determine efficacy of enzymatic cleavage of [^18^F]2, PLE (4.8 U dissolved in 1 mM HEPES) was added to 3 MBq [^18^F]2 and incubated at 37 °C, with aliquots taken at 5, 10 and 20 min. Heat inactivated PLE (95 °C for 1 h) was used as a control. At indicated timepoints, samples were precipitated with ice cold methanol, centrifuged at 4 °C at top speed, and the supernatant diluted for HPLC.

### Western blotting

Western blot analysis was carried out using an iBind Flex system (ThermoFisher Scientific) for primary and secondary antibody immunoblotting following a previously described method.^29^ For cell lysate collection SKOV3-ip1 and SKOV3-TRip2 cells were seeded in 6-well plates for 24 h at a density of 1.25 × 10^5^ mL^−1^ and 1.75 × 10^5^ mL^−1^, respectively, in 2 mL of media. Lysates were collected in RIPA buffer, protein quantified by Pierce BCA assay and 20–30 μg loaded on a 10% polyacrylamide gel which was run at 200 V. Rabbit monoclonal primary antibodies against anti-ALDH1A1 (Cell Signaling Technology) was used at a concentration of 1 : 1000 and anti-rabbit IgG secondary antibody (1 : 200 dilution; Cell Signaling Technology) for cell lysates analysis. Actin was used as a loading control for all experiments (1 : 1000 dilution; Cell Signaling Technology). After antibody incubations membranes were washed 5× with tris-buffered saline with tween (TBST) and visualised using ECL regent (GE Healthcare), with images taken using an iBright CCD camera (Invitrogen). Images were always acquired within the linear range of the camera to prevent the overexposure of any blots.

### Cell uptake

For cell uptake studies SKOV3-ip1 and SKOV3-TRip2 cells were plated in 6 well plates 24 h prior to the studies, as described above. For treatment with DEAB, a final concentration of 30 μM was administered 15 min prior to radiotracer uptake and remained in the media throughout the uptake time course. One mL of fresh media containing 0.37 MBq of [^18^F]2 was added to each well for the desired incubation time. At 20 min, plates were placed on ice, washed three times with ice-cold PBS to remove exogenous radioactivity and lysed in RIPA buffer (500 μL; Fisher Scientific Ltd). Decay-corrected radioactivity in lysates was determined on a gamma counter (300 μL of lysate; Wallac 1282 CompuGamma γ counter), with the remaining cell lysate centrifuged (21 000 × *g* for 10 min at 4 °C) and the supernatant used to determine protein concentration following radioactive decay using a Pierce BCA assay. To quantify radiotracer uptake in cells, three standard solutions of the radioactivity-containing medium were counted on the gamma counter. Data were expressed as a percentage of total radioactivity administered to cells per mg of protein. Molar activity studies were conducted by adding [^19^F]2 to achieve a range of molar activities.

### Blood metabolite analysis

Blood metabolism analysis of [^18^F]2 was performed by radio-HPLC. The amount of parent radiotracer and corresponding metabolites were quantified based on the area under the curve for [^18^F]2 (retention time 18.07 min) and its corresponding metabolites: [^18^F]1 (retention time 13.35 min), [^18^F]3 (retention time 6.31 min) and [^18^F]4 (retention time 12.10 min), and expressed as a percentage of total radioactivity (mean ± SD).

Blood was harvested using a syringe containing heparin and added to a heparinised tube. [^18^F]2 (1.0 MBq, 5–10 μL) was added to approx. 500 μL of blood before immediate incubation at 37 °C. Samples (150 μL) were taken for processing at 2, 5 and 10 minutes. Blood samples were centrifuged (2000 × *g* for 5 min, 4 °C), the plasma was removed and transferred to another protein low-bind Eppendorf. Ice-cold MeOH (500 μL) was added to the plasma and the sample briefly mixed on a Vortex. Samples were then centrifuged (12 000 × *g* for 5 min, 4 °C), and the supernatant transferred to a glass vial through a Millex 0.2 μm filter (Millipore, Billerica, MA, USA). The samples were diluted with 1 mL aqueous ammonium acetate (10 mM), passed through another Millex 0.2 μm filter (Millipore, Billerica, MA, USA) and analysed by reverse phase HPLC (ZORBAX® StableBond 300 C18, 9.4 × 250 mm, 5 μm HPLC column at room temperature; solvent A: H_2_O (10 mM NH_4_OAc), solvent B: MeOH; flow rate: 3.5 mL min^−1^; UV detector: 254 nm; gradient: 30–58% B, 0–14 min; 58–95% B, 14–21 min; 95% B; 21–27 min; 95–37% B, 27–28 min, 2.0 mL injection loop).

### *In vivo* tumour models

All animal experiments were performed in accordance with the United Kingdom Home Office Animal (scientific procedures) Act 1986. 2 × 10^6^ SKOV3-ip1 and 4 × 10^6^ and SKOV3-TRip2 cells were injected subcutaneously into female Balb/c nu/nu mice aged 6–9 weeks (Charles River Laboratories). Tumour dimensions were measured using an electronic calliper and the volume calculated using the following equation: volume = ((π/6) × *h* × *w* × *l*), where *h*, *w* and *l* represent, height, width and length, respectively. Imaging studies took place when tumour size reached approximately 150–250 mm^3^.

### PET imaging

Dynamic PET scans were acquired on a Mediso NanoScan PET/CT system (1–5 coincidence mode; 3D reconstruction; CT attenuation-corrected; scatter corrected). Mice received a bolus intravenous injection of approximately 3.0 MBq of [^18^F]2 through a tail vein cannular on a four mouse imaging bed.^30^ A 60 min PET scan was acquired immediately after the injection of the radiotracer. Animals were maintained under isoflurane anaesthesia (1.5–2% in oxygen) at 37 °C during and after radiotracer administration, and throughout the scan. CT images were acquired for anatomical visualisation and for CT attenuation correction (480 projections; helical acquisition; 50 kVp; 300 ms exposure time).

The acquired data were sorted into 19 time frames of 4 × 15 seconds, 4 × 60 seconds, and 11 × 300 seconds for image reconstruction (Tera-Tomo 3D reconstructed algorithm; 4 iterations; 6 subsets; 400–600 keV; 0.3 mm 3 voxel size). VivoQuant software (v.2.5, Invicro Ltd.) was used to analyse reconstructed images. Regions of interest were drawn manually using CT images and 50–60 min summed dynamic PET images as reference. Time *versus* radioactivity curves (TACs) were generated using the percentage injected dose per mL (% ID mL^−1^) and from this the area under the time *versus* radioactivity curve (AUC) was generated.

### Statistics

All statistical analysis was performed using GraphPad Prism (v.8.0) on only data sets acquired from three or more biological replicates, acquired on separate days. The data shown were expressed as the mean ± one standard deviation (SD). Statistical significance was determined using unpaired Student's *t*-tests or ANOVA (Tukey's multiple comparisons), with data considered significant if *P* < 0.05.

## Funding

This study was funded through a Wellcome Trust and Royal Society Sir Henry Dale Fellowship (107610/Z/15/Z) and a Wellcome Trust Senior Research Fellowship (220221/Z/20/Z) to THW. As this research was funded by the Wellcome Trust and for the purpose of open access, the author has applied a CC BY public copyright licence to any Author Accepted Manuscript version arising from this submission.

## Conflicts of interest

THW has received commercial funding from GlaxoSmithKline and Life Molecular Imaging. The other authors have no conflicts of interest to declare.

## Supplementary Material

CB-003-D2CB00040G-s001
