# Supplementary material for: A prodrug strategy for the in vivo imaging of aldehyde dehydrogenase activity
Source: RSC Chem Biol. 2022 Mar 11;3(5):561–70. doi: 10.1039/d2cb00040g (PMC9092432; doi:10.1039/d2cb00040g)

Electronic Supplementary Material (ESI) for RSC Chemical Biology.
This journal is © The Royal Society of Chemistry 2022

A Prodrug Strategy for the *In Vivo* Imaging of Aldehyde Dehydrogenase Activity

SUPPLEMENTARY INFORMATION

Raul Pereira*, Renée L. Flaherty*, Richard S. Edwards*, Hannah Greenwood, Adam J Shuhendler, Timothy H. Witney

[1] School of Biomedical Engineering and Imaging Sciences, King's College London, St Thomas' Hospital, London, SE1 7EH

[2]Department of Chemistry & Biomolecular Sciences, University of Ottawa, Ottawa, ON, Canada and University of Ottawa Heart Institute, Ottawa, ON, Canada

*Contributed equally

[†]Corresponding author: Timothy H. Witney (tim.witney@kcl.ac.uk); Tel: +44 (0)20 7188 7188 ext. 883496; Address: School of Biomedical Engineering & Imaging Sciences, King's College London, St Thomas' Hospital, London SE1 7EH

TABLE OF CONTENTS

Supplemental Figures……………………………………………………………………………………………………S2

Supplemental Tables……………………………………………………………………………………………………S6

Spectra……S7

SUPPLEMENTAL FIGURES

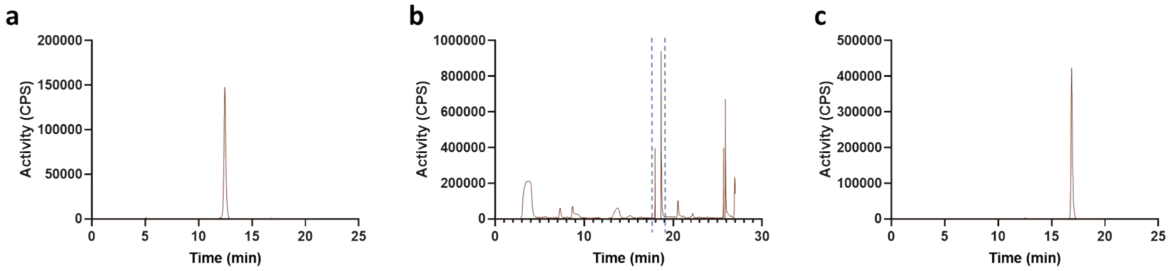

Supplementary Fig. S1. *Example analytical and semi-preparative chromatograms from the synthesis of **[^{18}F]1** and **[^{18}F]2** (HPLC conditions A). a) Analytical radio-chromatogram of **[^{18}F]1** following its automated synthesis on the GE FASTlab^{TM}. b) Radio-chromatogram showing the isolation of **[^{18}F]2** by semi-preparative HPLC after it's manual radiosynthesis. Blue dashed line indicate the collected fraction. Due to the large amounts of radioactivity present, peaks have reached the saturation threshold of the detector, giving the familiar U shaped peaks seen here. c) Analytical radio-chromatogram of **[^{18}F]2** following semi-preparative HPLC purification and reformulation in PBS.*

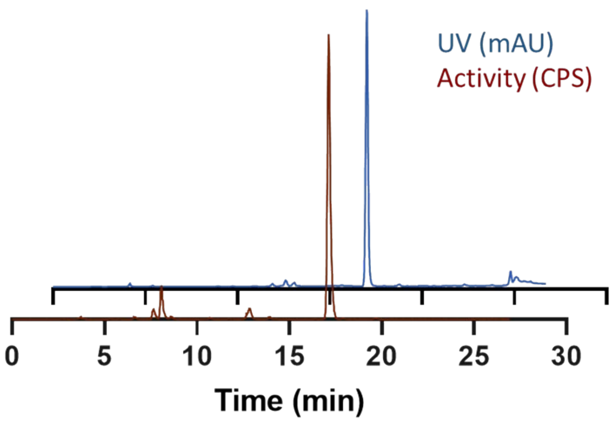

Supplementary Fig. S2. *Identification of **[^{18}F]2** (HPLC conditions A). Co-injection of 2 (retention time 17.01 min, UV) with the crude reaction mixture for the manual synthesis of **[^{18}F]2** (retention time 17.13 min, activity).*

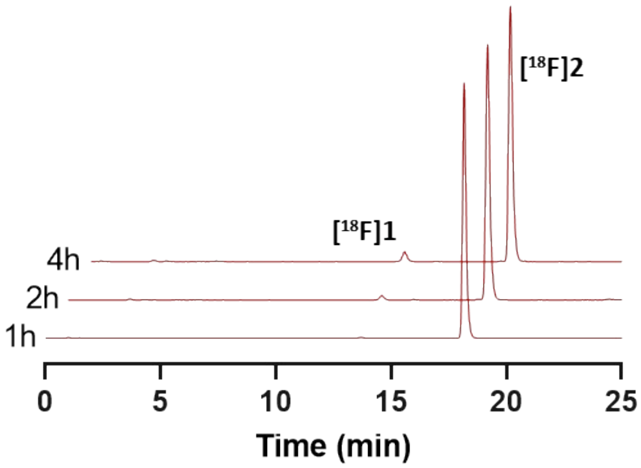

Supplementary Fig. S3. Formulation stability of [18F]2. Example chromatograms of [18F]2 in PBS over time at room temperature (HPLC conditions A). Slow hydrolysis to [18F]1 is observed. However, radiochemical purity at 4 h remained >95% (n = 3).

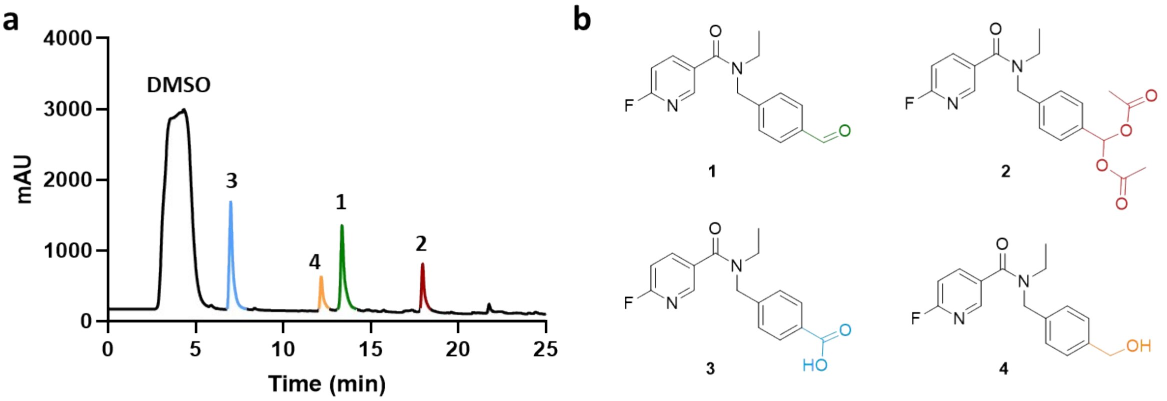

Supplementary Fig. S4. Identification of the metabolites of 2. *a) Chromatogram showing the retention times of the ^{19}F 'cold' standards; **1** (retention time 13.37 min), **2** (retention time 17.97 min), **3** (retention time 7.01 min) and **4** (retention time 12.18 min). b) Chemical structures of the ^{19}F 'cold' standards; **1**, **2**, **3** and **4**. Compounds **1**, **3** and **4** were prepared as previously described [21].*

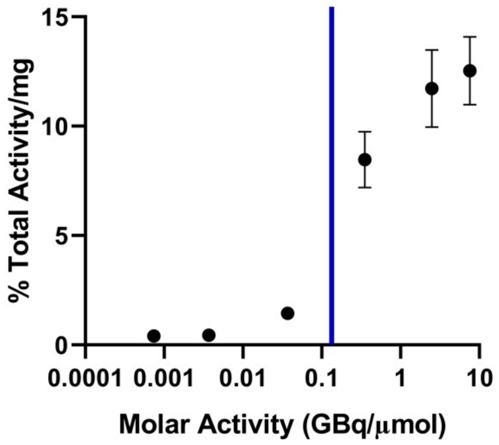

Supplementary Fig. S5. *Effect of molar activity on [^{18}F]**2** cell uptake. SKOV3-TRip2 cells were co-incubated with [^{18}F]**2** and various concentrations of 2 for 20 min. The blue line shows the chosen molar activity to use for low molar activity experiments (0.11 GBq/µmol). Data are mean values ± SD of three biological replicates.*

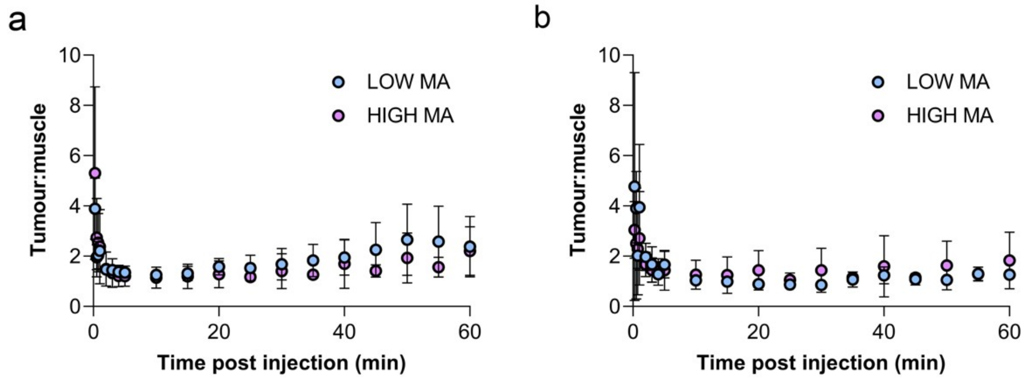

Supplementary Fig. S6. *Tumour to muscle time activity curves of mice bearing SKOV3-ip1 (a) or SKOV3-TRip2 (b) subcutaneous tumours at low and high MAs.*

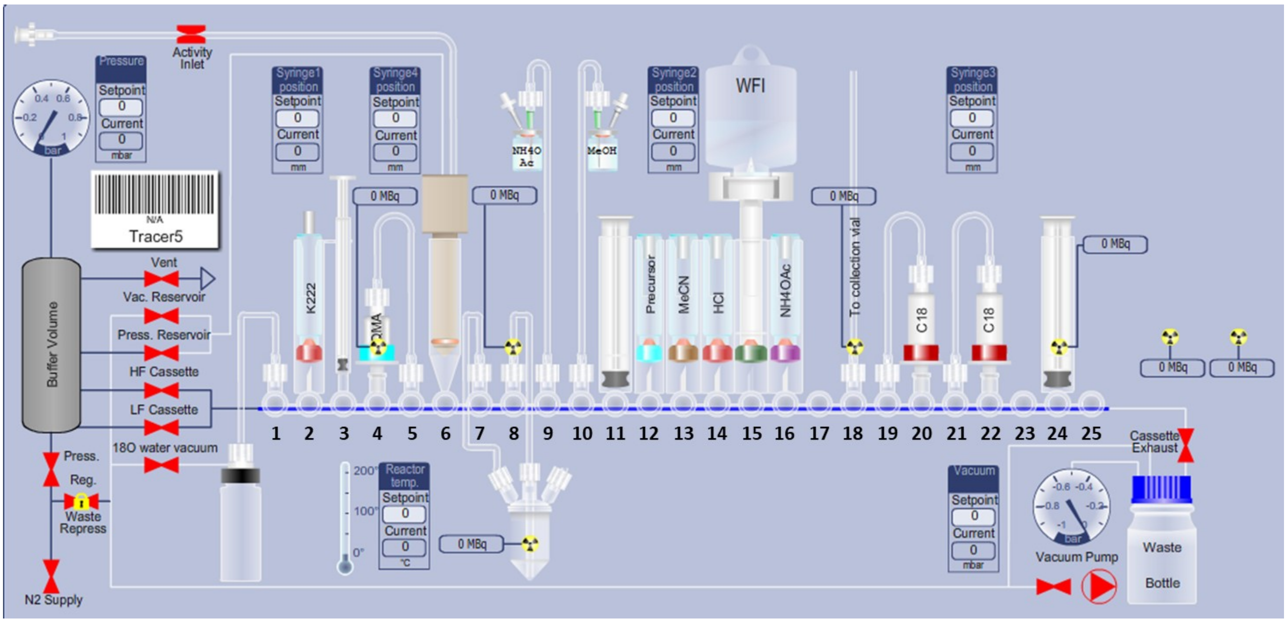

*Supplementary Fig. S7. Cassette layout for the synthesis of [^{18}F]**1**. Numbers refer to each individual valve or cassette position (CP). Consumables and reagents added to each position are described in **Supplementary Table 1**. Tubing from the reactor to CP 25 is not shown for clarity. WFI, Water for injection.*

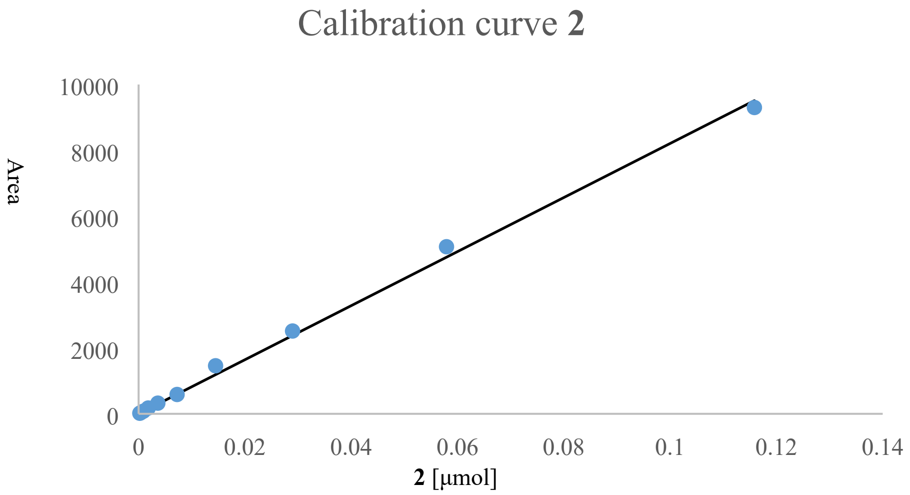

Supplementary Fig. S8. *HPLC UV calibration curve used to calculate the molar activity of [^{18}F]**2**.*

SUPPLEMENTAL TABLES

Supplementary Table 1. FASTlab cassette reagent positions for the radiosynthesis of [^{18}F]**1**.

Cassette Position(CP)	Reagent, hardware or consumable
1	Short tubing to ^{18}O water collection vial
2	Kryptofix® carbonate solution (11 mm Vial, 850 µL)
3	Syringe 1
4	QMA light SepPak cartridge
5	Short tubing to QMA light SepPak cartridge at CP4
6	^{18}F Inlet
7	Short tubing to reactor (LHS)[a]
8	Short tubing to reactor (Center)
9	Long tubing to external vial (NH_4OAc, 30 mL, 20 mM)
10	Long tubing to external vial (MeOH, 25 mL)
11	Syringe 2
12	Precursor Solution (11 mm Vial, 1.0 mL)
13	Dry acetonitrile (13 mm Vial, 1.6 mL)
14	1M HCl (13 mm Vial, 1.0 mL)
15	Water Spike/ water bag
16	20 mM NH_4OAc (13 mm Vial, 3.0 mL)
17	Unused
18	Long tubing to collection vial
19	Short tubing to C18 SepPak cartridge at CP20
20	C18 SepPak cartridge
21	Short tubing to C18 SepPak cartridge at CP22
22	C18 SepPak cartridge
23	Unused
24	Syringe 3
25	Long tubing to reactor (RHS)[b]

[a] LHS = Left hand side, [b] RHS = Right hand side.

SPECTRA

^{1}H NMR (400 MHz, DMSO-$_{d6}$, 298K) of (4-((*N*-ethyl-6-fluoronicotinamido)methyl)phenyl)methylene diacetate **2**

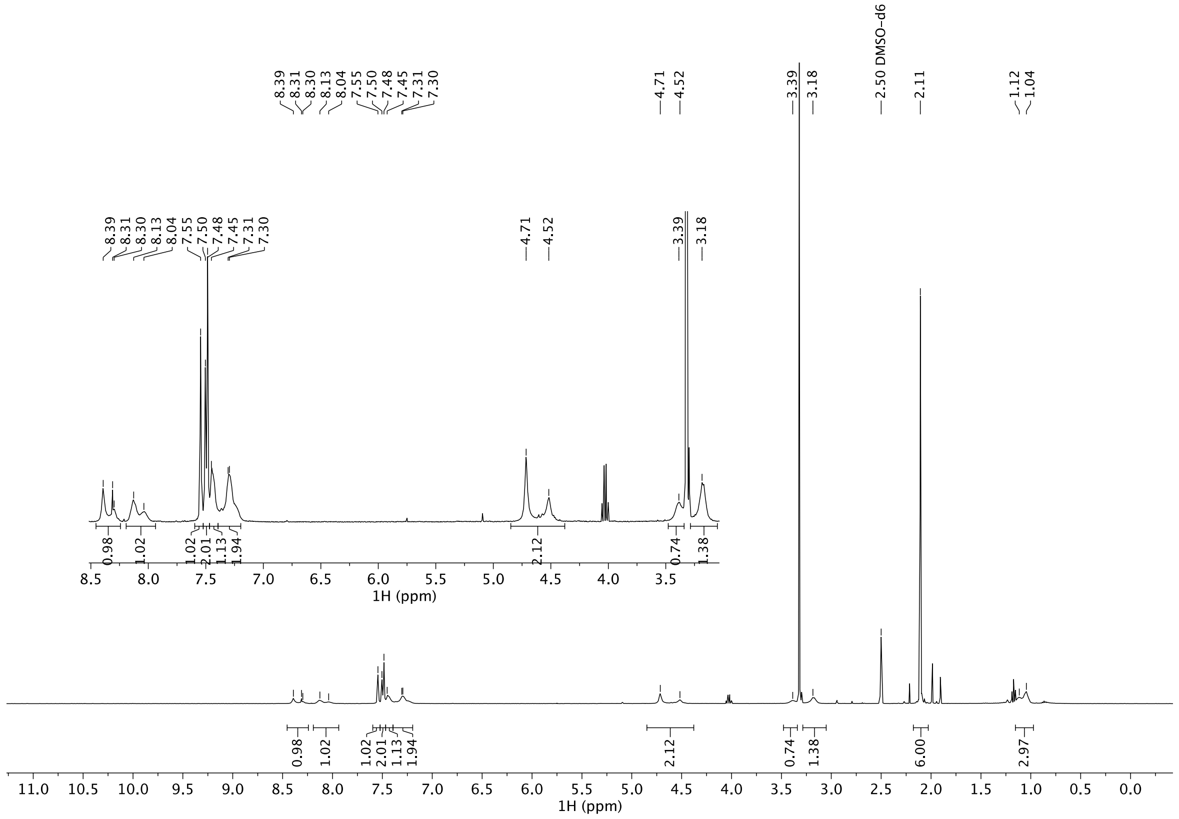

^{13}C NMR (100 MHz, DMSO-$_{d6}$, 298K) of (4-((*N*-ethyl-6-fluoronicotinamido)methyl)phenyl)methylene diacetate **2**

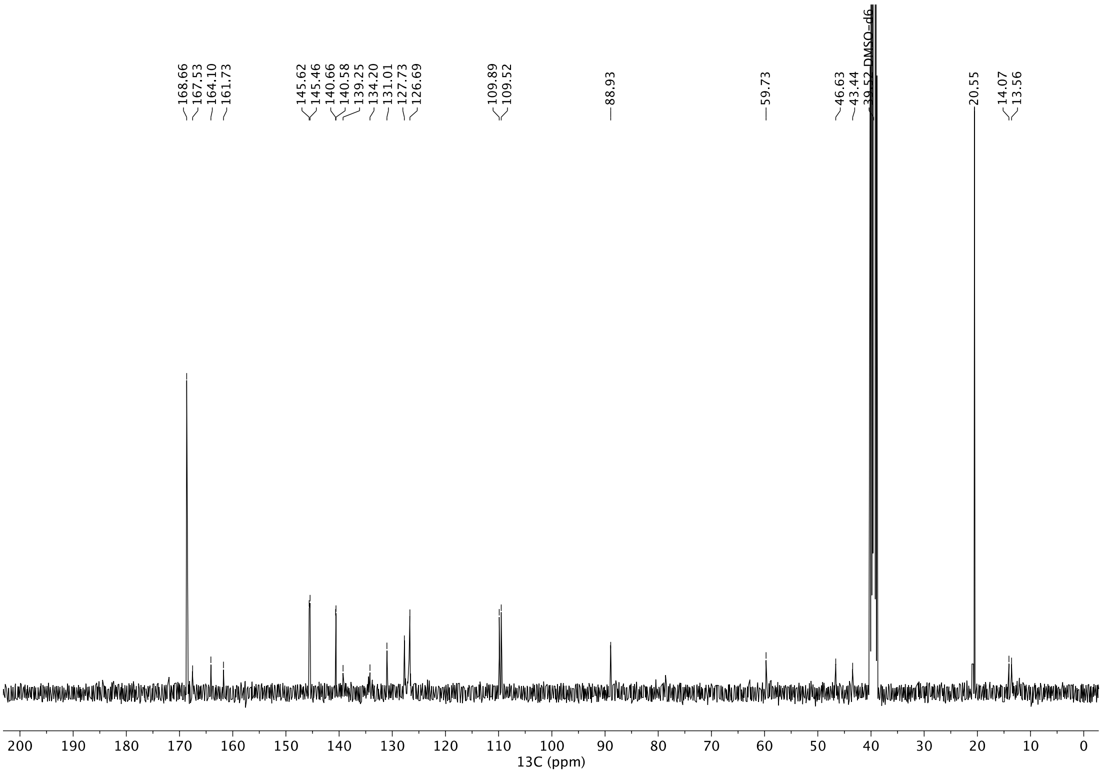

^{19}F NMR (376 MHz, DMSO-$_{d6}$, 298K) of (4-((*N*-ethyl-6-fluoronicotinamido)methyl)phenyl)methylene diacetate **2**

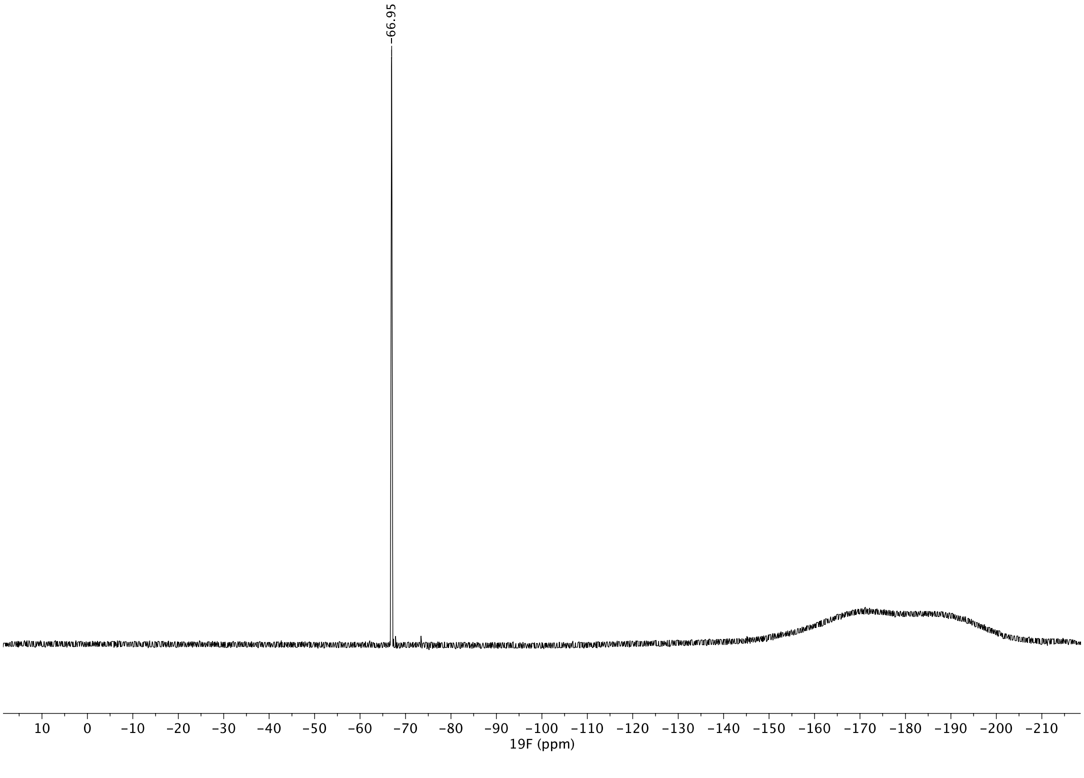

HRMS of of (4-((*N*-ethyl-6-fluoronicotinamido)methyl)phenyl)methylene diacetate **2**

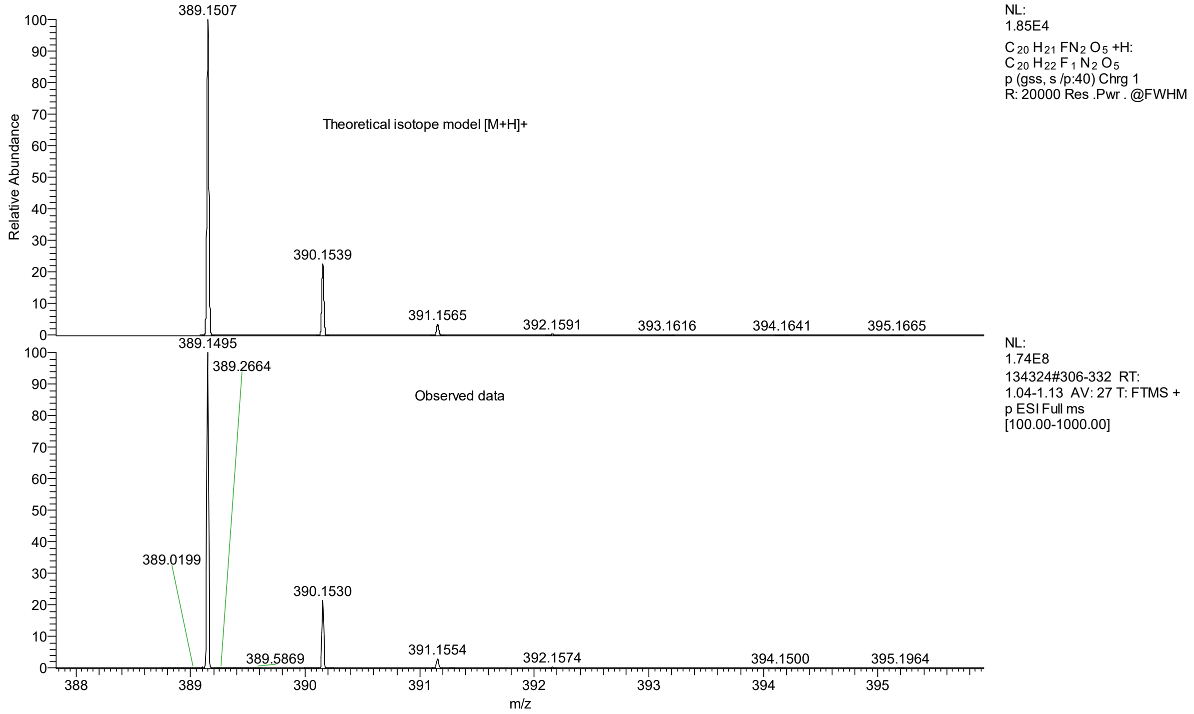

Supplement: CB-003-D2CB00040G-s001 [file CB-003-D2CB00040G-s001.pdf]
